# National focal points and implementation of the International Health Regulations

**DOI:** 10.2471/BLT.20.270116

**Published:** 2021-05-04

**Authors:** Kumanan Wilson, Sam Halabi, Helge Hollmeyer, Lawrence O Gostin, David P Fidler, Corinne Packer, Lindsay Wilson, Ronald Labonté

**Affiliations:** a Department of Medicine, Ottawa Hospital, Civic Campus, 1053 Carling Avenue, Box 684, Administrative Services Building, Ottawa, ON K1Y 4E9, Canada.; b O'Neill Institute for National and Global Health Law, Georgetown University, Washington DC, United States of America (USA).; c World Health Organization, IHR Secretariat, Geneva, Switzerland.; d Council on Foreign Relations, New York, USA.; e School of Epidemiology and Public Health, University of Ottawa, Ottawa, Canada.; f Global Strategy Lab, York University, New York, USA.

As the coronavirus disease 2019 (COVID-19) pandemic continues, the World Health Organization (WHO), the International Health Regulations (IHR) and countries’ adherence to IHR guidance are coming under scrutiny and review.^1^^,^^2^ The IHR constitute a legal and governance framework that guides countries in responding to serious disease events while avoiding unnecessary interference with international trade and traffic.^3^ The IHR require States Parties to designate or establish national IHR focal points to facilitate information sharing about disease events with WHO, which makes these focal points critical in the effective implementation of the IHR within and between countries. On behalf of the State Party concerned, national focal points are responsible for timely notification to WHO of relevant health events, responding to WHO Secretariat requests for event-related information, and ensuring that messages and advice from WHO are disseminated to the relevant sectors within the country. 

A review of the 2013–2016 Ebola virus disease outbreak in West Africa found deficiencies in the functioning of national focal points.^4^ Published studies have also identified technical and political challenges to the notification of events by focal points to WHO.^5^^,^^6^ At the request of WHO, we evaluated the ability of focal points to carry out their IHR functions through 25 in-depth interviews and 105 online quantitative surveys. Here we present summary findings and recommendations emerging from our study; survey methods and results have been previously published.^7^

## Main observations

We found that most national focal points are aware of their duties and responsibilities under the IHR. Furthermore, we did not find evidence of intentional non-compliance with the IHR, although some focal points reported concerns as to how WHO may use the information provided when reporting events. While national focal points reported sufficient knowledge about their IHR obligations, some expressed uncertainty over how to report a public health event.

Focal points reported that, should a public health event occur, they know who to contact at the WHO regional level and have the ability to send urgent event-related communications to WHO. However, focal points identified weaknesses in communications that adversely affect their functioning. For instance, some reported that their offices are not accessible at all times for urgent communications to WHO. Others indicated that they do not have the appropriate information technology to carry out the assigned communication functions. By contrast, respondents described quite robust communications with other focal points in different countries and indicated that their governments are favourable to strengthening peer-to-peer communications if WHO would develop and oversee a national focal point-focused learning and sharing network.

While focal points are aware of States Parties obligations under the IHR, internal challenges exist in fulfilling these obligations. Many focal points must obtain approval from one or more governmental authorities outside the health sector – many of whom are not familiar with the IHR – before they can notify WHO of disease events. Focal points indicated that colleagues in these related sectors have an insufficient understanding of the role of national focal points or of how and when to engage with them.

The intersectoral approval process can include other ministries scrutinizing the accuracy of information and documents, and other departments at the national level evaluating the potential adverse impact of a notification. Focal points repeatedly identified this scrutiny as a challenge, along with the lack of understanding in other government agencies about the IHR and not having access to the relevant ministries and decision-makers. These factors can jeopardize the timeliness of information sharing and the expeditious reporting of public health events to WHO, as many focal points indicated that they cannot proceed to issue a notification to WHO without first receiving clearance from decision-makers in these other sectors.

Difficulties in communicating information between focal points and other sectors in their government compound the challenge of obtaining intersectoral approval. To perform its functions and submit reports to WHO in a timely fashion, the office of the national focal point depends on input from other ministries and agencies in related sectors outside of health. Respondents reported challenges in their ability to disseminate information from WHO to relevant sectors in their countries and to consolidate input from these sectors in a timely fashion. Some national focal points believed that existing communication procedures and structures in their countries are not sufficient to ensure timely and effective communication between themselves and national stakeholders in other sectors.

For focal points to be able to carry out their functions, adequate training and ongoing learning opportunities need to exist. WHO offers many information and training resources to national focal points, including the *National IHR focal points guide*, tutorials and guidance on the use of Annex 2 of the IHR, the *Toolkit for implementation in national legislation* (an online IHR training toolkit course) and access to knowledge networks and regional workshops. However, many national focal points lack awareness of these resources. For those aware of the tools, many commented that their format could be improved by, for example, permitting offline digital learning.

Furthermore, half of the 105 States Parties surveyed reported having no plan to support the continuous development and learning of staff of the national focal points, which is cause for concern. In addition, national focal points reported that the general nature of WHO’s materials makes them less useful for specific instances such as chemical, radiation and nuclear events. Some focal points suggested that these events were neglected or peripheral to infectious disease events in the IHR guidance. Many identified that more financial resources, equipment and technological support would allow them to perform their IHR functions better. They reported that turnover and absences among staff, often due to human resources constraints, make continuity of functions challenging, a problem that heightens the need to train new staff rapidly on the IHR and the functions of a national focal point. Some also described inadequate staffing of offices.

## Recommendations 

For WHO to further support national focal points in the challenges we have identified, the most feasible and rapid solutions centre on improving WHO training materials and tools for, and focusing more attention on, supporting the focal points’ efforts to integrate training into their standard operating procedures. These improvements would include updating the *National IHR focal points guide*, making it available in more languages, improving the content and accessibility of online training tools and increasing awareness of the training resources. 

Several focal points mentioned the value of peer-to-peer communication to assist with carrying out IHR functions. Given how IHR tasks may be very specific to local circumstances – something that can be difficult to capture in general guidance – support from peers in how to handle unique local issues can be valuable. Thus, raising further awareness of WHO’s IHR Event Information Site for National IHR Focal Points, a secure website developed and maintained by WHO’s Secretariat to provide all States Parties with information about acute public health events, and providing further support of peer-to-peer communications would be helpful. 

Over the intermediate term, WHO should emphasize the importance of States Parties meeting core capacity requirements to detect, assess, report and respond to public health events. Doing so could assist focal points in obtaining support to perform their functions. WHO could also share best practices for national focal points to address three identified governance challenges: first, how to execute functions in the absence of the national focal points’ legal authority over other sectors; second, strategies to expedite obtaining approval from other ministries; and third, approaches to address competing political and economic considerations that could impact reporting of public health events. Participants acknowledged that these challenges are a result of their own governance structures and internal hierarchies, but they still requested assistance from WHO on how to navigate these issues. Suggestions for WHO support included sharing governance approaches (such as memoranda of understanding, interministerial agreements and enabling legislation) and the perceived success of these approaches in facilitating the focal points’ ability to execute their functions.^8^

Also over the intermediate term, WHO could provide further support to intersectoral collaboration challenges. National focal points are responsible for disseminating information to, and consolidating input from, relevant sectors of the administration of the State Party concerned.^3^ This task includes helping to build IHR knowledge and capacity in ministries outside of health so that States Parties are better able to implement the IHR. To do so, WHO could collect and share best practices and provide guidance for intersectoral communication and collaboration and establishing communication protocols between ministries. Additionally, WHO could also share best practices on raising awareness across sectors of the importance of providing national focal points with the necessary authority when notifications of public health events must be approved and issued. WHO’s approach in addressing these specific issues must carefully navigate issues around state sovereignty.

Many of our findings reflect those of previous reports on the IHR including the need for intersectoral collaboration, problems related to high turnover of personnel in the national focal point office and general need for resourcing.^4^ While our analysis was conducted just before the COVID-19 pandemic, determining whether any of our identified barriers played a role in the global response to the pandemic will be important. Particularly, questions have arisen about whether reporting was timely and comprehensive among States Parties.^9^ In the post-pandemic review of the IHR, it will be important to determine what role the barriers we identified to the national focal points’ ability to execute their functions may have played. A statement from the Chair of the IHR Review Committee underscoring the importance of empowering national focal points and ensuring they are integrated into the emergency decision-making process was consistent with our studies’ findings.^10^ We expect comprehensive approaches to strengthening and supporting national focal points and raising awareness of the IHR across all relevant sectors of government will emerge as priorities to prepare for future public health emergencies.
